# First-Stage Algorithm for Photo-Identification and Location of Marine Species

**DOI:** 10.3390/ani16020281

**Published:** 2026-01-16

**Authors:** Rosa Isela Ramos-Arredondo, Francisco Javier Gallegos-Funes, Blanca Esther Carvajal-Gámez, Guillermo Urriolagoitia-Sosa, Beatriz Romero-Ángeles, Alberto Jorge Rosales-Silva, Erick Velázquez-Lozada

**Affiliations:** 1 Instituto Politécnico Nacional, Escuela Superior de Ingeniería Mecánica y Eléctrica, SEPI-ESIME, Av. IPN s/n, Col. Lindavista, Ciudad de México 07738, Mexico; 2 Instituto Politécnico Nacional, SEPI-UPIITA, Av. IPN 2580, Ciudad de México 07340, Mexico

**Keywords:** photo-identification, animal location, BMG, GMR

## Abstract

Marine species photo-identification and location for tracking are crucial for understanding the characteristics and patterns that distinguish each marine species. However, challenges in camera data acquisition and the unpredictability of animal movements have restricted progress in this field. To address these challenges, we present a novel algorithm for the first stage of marine species photo-identification and location methods. For marine species photo-identification applications, a color index-based thresholding segmentation method is proposed. This method is based on the characteristics of the *GMR* (Green Minus Red) color index and the proposed empirical *BMG* (Blue Minus Green) color index. These color indexes are modified to provide better information about the color of regions, such as marine animals, the sky, and land found in the scientific sightings images, allowing an optimal thresholding segmentation method. In the case of marine species location, a *SURFs* (Speeded-Up Robust Features)-based supervised classifier is used to obtain the location of the marine animal in the sighting image; with this, its tracking could be obtained. The tests were performed with the Kaggle happywhale public database; the results obtained in precision shown range from 0.77 up to 0.98 using the proposed indexes. Finally, the proposed method could be used in real-time marine species tracking with a processing time of 0.33 s for images of 645 × 376 pixels using a standard PC.

## Simple Summary

Scientific sighting images are acquired during the time marine species emerge to the sea surface and until they submerge again. During this time, marine biologists manually make and capture their observations related to the abundance, conservation, and behavior of different species. This is inefficient and error-prone in many analysis scenarios. Smart cameras are commonly used to acquire the marine animal image and then process this image using computer vision systems to develop different applications. In this paper, a novel algorithm for the first stage of marine species photo-identification and location methods is presented. A color index-based thresholding method is proposed as a segmentation method that can be used as the first stage in photo-identification applications, and a *SURFs* (Speeded-Up Robust Features)-based method is proposed to obtain the location of marine animals in the image, which can be used as the first stage in marine species tracking methods. Finally, the proposed scheme is simple, efficient, and feasible for photo-identification and tracking applications.

## 1. Introduction

Marine biologists realize scientific sightings for the study of different marine species, among which are large species such as cetaceans [1]. In scientific sightings, samples of organic material are collected and digital images of the species to be studied are acquired [2]. Several countries have regulated the sighting of marine species for scientific and ecotourism purposes, allowing digital images to be captured from a distance and in conditions where the behavior of the marine animal is not altered [3]. Digital cameras with 300 mm lenses can capture images with sufficient detail of the areas where the characteristics to be identified of the marine animal under study are located at the distance regulated by each country [2,4]. In addition, the capture information must be the same for different sightings, complying with aspects of quality in exposure and composition [1,2,3,4,5,6].

The photo identification and location for tracking of marine species are two applications where the scientific community in the marine study area has focused efforts to minimize classification bias and save time in classifying the species studied.

In photo-identification of marine species, the information collected from digital images of scientific sightings is crucial for the identification of characteristics such as the dorsal and caudal fins, smooth hairless skin, nostrils in the head, pigmentation in the skin, markings, spots and shapes on the skin, movement patterns, individual behavior, and population size that distinguish each marine animal [1,2,3,4]. Digital image processing is commonly used as a non-invasive and inexpensive technique. For this reason, computer-aided systems are used to improve the images obtained and provide a better photo identification [5,6,7,8,9,10,11,12]. The images must achieve the criteria for deciding whether they are useful for photo identification [12]. The criteria used to discard an image are the following:

1. The content of the image contains elements unrelated to the marine species to be studied, the number of animals in the captured image, or the image has no sufficient representative elements for its use;
2. The image acquired can be affected by lighting, blurring, distortion, exposure level, focal length, and others.

Animal location for tracking [13] is a technology used in ecology, zoology, and environmental science. This provides insights into wildlife behavior and conservation, migration patterns, movement trajectories of animals, territory utilization, prediction of disease spread, behavior of animals, and the health of ecosystems. In the case of marine species tracking, smart cameras are utilized to acquire the marine species video and then process the video using computer vision systems to track the specific animal targets [14]. In animal behavior studies, computer vision methods are used for analyzing and predicting patterns in large and complex datasets and provide a detailed understanding of movement, interactions between species, and overall health [15]. Marine species tracking provides a non-invasive and inexpensive technique, avoiding the installation of sensors on the animals to pick up data. This captures data for all animals within the camera’s field of view, reducing the cost associated with the data acquisition. On the other hand, animal tracking methods present different issues due to the unfavorable environmental conditions when the video data is acquired [14].

Currently, it is difficult to find a technique that brings together all these characteristics due to the complexity presented during the monitoring and acquisition of images of this species. There are studies that implement machine learning-based techniques, such as the one cited below: Sanikommu et al. [16] present a technique based on multiple machine learning architectures, such as U-Net, YOLO v8, and Resnet101, which yields an accuracy of up to 98%. This work requires high-resolution satellite images to obtain the results, as well as a complex computing architecture. Another example is presented by Pollicelli et al. [17], who present a dolphin tracking system using the dorsal fin. The method used is Mask R-CNN, which is based on Resnet101. This work applies the convolutional network for image segmentation, comparing it with a manually segmented image to find the points of intersection. The results table shows values between 0 and 99.2% for tracking dolphins. The work relies directly on a manual segmentation template to track the dolphin. A machine learning-based method implemented in photo-identification in animals using efficientnet was also investigated. This method is primarily used when there are few images available for identification. An example of application is given by Rahman Oion et al. [18], who present a result in the identification of various marine animals, achieving an accuracy of 87.99%. This method, like the previous one, uses manual segmentation to perform the classification and thus the identification. Another method used is the Siamese Net; an example in the area of animal identification is the recognition of sheep faces. This work is presented by Zhang et al. [19], which achieves an accuracy between 86.6% and 92.1%. Finally, as a trend in machine learning and deep learning related to animal identification, we have Vision Transformers (ViTs). This type of method encompasses global patterns, requiring a large amount of data to produce a result. This is the example presented by Yang et al. [20]. The authors perform the classification considering the following: The model considers hierarchical levels encompassing 3 classes, 38 orders, 154 families, 438 genera, and 808 species. FishAI achieved accuracies of 0.975 (class), 0.798 (order), 0.743 (family), 0.638 (genus), and 0.626 (species) in the test images, respectively, through hyperparameter optimization. An example for YOLOv5/YOLOv8 is the one implemented for the dorsal fin in dolphins, a work presented by Kim et al. [21]. The authors propose an architecture based on YOLOv8 with a result of 88% accuracy without previously applying the improvement also made by them.

While applying existing tools is tempting for use under real conditions and in real time, many potential pitfalls must be considered to ensure the responsible use of these approaches [22,23]. For example, the most popular method for marine species applications is the deep learning approach. This approach has been very successful, but the limitations of this approach include the need for a large amount of data (hundreds/thousands) to train these deep-learning models accurately [22,23]. However, because many species rarely occur, only a few shot samples are available; thus, the performance is typically low [22].

The contributions of this paper are shown below:

1. A novel algorithm for the first stage of marine species photo-identification and location methods is presented.
2. The proposed method can resolve the limitations (large training data) presented in the deep learning approaches.
3. The NGMR (Normalized Green Minus Red) and NBMG (Normalized Blue Minus Green) color indexes are proposed to provide better information about the color of regions (marine animal, sky, and land) found in the scientific sightings
4. The proposed method is simple, efficient, and feasible for use under real conditions and in real time in marine species applications. It does not require a lot of computing resources, and its implementation is straightforward on any device and could provide a real-time solution to process marine animal video frames.

The rest of this paper is structured as follows: Section 2 presents the proposed marine animal segmentation and location methods, including the color index-based thresholding method, the NGMR (Normalized GMR) and NBMG (Normalized BMG) color indexes, and the marine animal classification and location method based on the SURFs (Speeded-Up Robust Features) classifier. Section 3 describes the experimental results for the segmentation and location of marine animals. Section 4 shows the discussion of the experiments and results obtained during this research, and Section 5 concludes our work.

## 2. Materials and Methods

### 2.1. Proposed Method

In this section, the proposed method is presented. First, the dataset of sighting images used for training and evaluation of the proposed algorithm is shown. Second, the *GMR* color index is used to obtain color information from the regions contained in the image; this index is modified to provide more color information for these regions. We also present the empirical *BMG* (Blue Minus Green) color index to improve the results of *GMR* in some cases. Third, the segmentation method by thresholding is presented; this method is proposed to segment marine animals, sky, and land. Fourth, the location of the marine animal in the image is performed using a supervised classifier with the *SURFs* method.

#### 2.1.1. Step 1: The Image Dataset

The image dataset is obtained from the happywhale competition in Kaggle for the identification of the humpback whale, and among them are images of whales and dolphins [24]. The dataset is of marine animals that can be identified by the dorsal fin, their flanks, and some characteristic features. This is made up of 28 organizations and agreements with cruise ships that sail in sighting areas at a speed of 11 mph to obtain 50,000 whales of 30 types of species, giving a set of 150,000 images. The images of sightings are given in RGB (Red, Green, Blue) color format using a telephoto lens (minimum 100 mm or 100–400 mm) with dimensions from 90 × 80 up to 3600 × 5269 pixels and a weight of 3–5703 Kb. Normally, these images are acquired from 7:00 to 19:00 h. of the day; this interval of time provides different image sequences that show different changes in the intensities of colors of the images, known as white balancing. This effect permits demonstrating that the proposed algorithm is robust to changes in illumination. Also, the happywhale dataset consists of images collected in real, uncontrolled environments captured by researchers, tour operators, and citizen photographers in various geographic regions. As a result, the dataset exhibits high variability in acquisition conditions, including different weather conditions (clear skies, cloud cover, haze, or sun glare), sea states (variable waves, foam), capture distances (from close-up shots to distant views), observation angles (frontal, lateral, oblique), and levels of resolution and image quality. Furthermore, the dataset includes images taken both at the surface level and partially underwater, with variations in lighting and contrast characteristic of the marine environment. This diversity of scenarios increases the complexity of the problem and allows for evaluating the robustness of the proposed method against heterogeneous visual conditions, closer to real situations of monitoring and sighting of marine fauna. For each RGB color image, we separate its color channels in an independent way, and we apply the next steps of the proposed method.

#### 2.1.2. Step 2: Calculation of the Color Indexes

The *GMR* color index is implemented for the diagnosis of nutrients in crops; that is, by detecting the green color of the leaves, the chlorophyll content and the percentage of nitrogen can be calculated [25,26]. The reflectance of the green color of vegetation has a high reflection peak compared to the color reflectance of other elements such as water and soil, so the *GMR* allows for better perception of the color differences between the elements of vegetation, soil, and water [26],

(1) GMR=G−R

where *G* and *R* represent the green and red channels, respectively.

The *GMR* provides a range of values of [−255, 255] according to all possible values of *G* and *R*. Some measures (e.g., Euclidean distance) implicitly assign more weight to the characteristics of components (parameters) with large ranges than those with small ranges [26]. This is also true in the *GMR* measure. In practice, several types of sighting images have different *GMR* values within the range described above, which can cause a fixed threshold method to not work efficiently. For this reason, feature normalization is required to equalize the ranges of the color index and make them have almost the same effect on the similarity calculation [26,27]. The goal is to independently normalize the characteristics of each component in Equation (2) to the range of [0, 1] using a normalization procedure from the linear scale to the range unit [27]. Therefore, the proposed *NGMR* (*Normalized GMR*) index is computed as,

(2) $$NGMR=\frac{\left(GMR+L\right)}{2L}$$

where *L* = 255 represents the maximum value of intensity in the image.

Taking into account the characteristics of the *GMR* color index, we propose an empirical index based on the calculation of the *B* channel minus the *G* channel (*BMG*). *BMG* can be used in images that highlight the blue and green colors, such as marine animal images, ocean or water surface images, and sky images. The *BMG* considers the appearance of the colors found in these images (i.e., azure, blue-gray, dark blue, light blue-green, and gray colors) [7]. In other words, the calculus of the blue channel minus the green channel provides information about different combinations of blue and green hues that, due to the short distance between these color ranges, helps to make the discrimination between marine animals, sea, and sky that are primarily found in sighting images. This new index has proved in several experimental results in sighting images to obtain good results; this can be computed as follows,

(3) BMG=B−G

In the same way that the *GMR*, a similar procedure used to obtain the proposed *NGMR*, is realized for the new *NBMG* (*Normalized BMG*), this is calculated as,

(4) $$NBMG=\frac{\left(BMG+L\right)}{2L}$$

where *L* = 255 is the maximum intensity value in the image.

Both indices help to obtain information about sea, land, marine animals, and sky regions in the sighting images. *NGMR* is computed as the normalized ratio of the mean gray-level intensity between adjacent regions. This metric captures global luminance contrasts that arise due to illumination conditions, atmospheric effects, and reflective properties of the observed surfaces. By normalizing the gray-level mean, *NGMR* becomes less sensitive to absolute brightness variations caused by camera settings or environmental lighting changes, which is particularly relevant in outdoor marine observations. On the other hand, *NBMG* is based on the normalized gradient of the blue channel mean values. *NBMG* emphasizes local chromatic transitions and edge information, which are critical for identifying the boundaries of marine animals against the surrounding environment.

#### 2.1.3. Step 3: Segmentation by Thresholding

From the happywhale database, the optimal thresholds are determined by using 100 images of each class, *Img_Cj_* (*Img_C_*_1_ = {100 images for land}, *Img_C_*_2_ = {100 images for marine animal}, and *Img_C_*_3_ = {100 images for sky}) as training images. The procedure to obtain the proposed thresholds is the following:

1. The *NGMR_Cj_* and *NBMG_Cj_* images for all *Img_Cj_* images of the three classes are computed;
2. Morphological operations of dilation and then erosion to all *NGMR_Cj_* and *NBMG_Cj_* images from Step 1 are realized to provide more uniform regions in intensity that correspond to the regions that make up a sighting image, obtaining the *NGMR_morphCj_* and *NBMG_morphCj_* images [28,29,30,31,32,33]. Simulations of morphological operations are carried out with different neighborhood sizes of 9 × 9, 10 × 10, 11 × 11, 12 × 12, 13 × 13, and 14 × 14 to find the optimal neighborhood size where the shape and the intensity of pigmentation in the skin of the marine animal are better preserved, and the uniformity of the different objects that make up the land and the sky. This is achieved using a neighborhood size of 11 × 11 pixels;
3. The histograms for all *NGMR_morphCj_* and *NBMG_morphCj_* images of each class are obtained, and a threshold for a determined class (land, marine animal, or sky region) of each histogram is found. The optimal thresholds are determined with the average of the thresholds obtained for each class (region). The segmentation thresholds for sky, land, and marine animals were calculated based on the variability of illumination presented in the sightings and the quality of the collected images, where automatic thresholding methods tend to be unstable. This proposal reduces the impact of noise and has a low computational cost. There exist other alternatives, such as automatic thresholding, unsupervised clustering, or deep learning-based segmentation, but these require unmet assumptions, big computational complexity, or unavailable volumes of labeled data. Therefore, the proposed approach is suitable for the objective of this work, which is to optimize resources in the detection of marine animals in heterogeneous images.

The segmentation by thresholding of any sighting image is achieved using the three optimal thresholds for a determined color index as follows,

(5) $$I_{segNGMR}=\left\{\begin{array}{cc} {NGMR}_{morph}\leq T_{NGMRC1}, & Land\\ T_{NGMRC2a}\leq \;{NGMR}_{morph}\;\leq T_{NGMRC2b}, & Marine\;animal\\ {NGMR}_{morph}\geq T_{NGMRC3}, & Sky \end{array}\right.$$

where *T_NGMRC_*_1_ = 0.4608, *T_NGMRC_*_2*a*_ = 0.0.4609, *T_NGMRC_*_2*b*_ = 0.5781, and *T_NGMRC_*_3_ = 0.5782 are the thresholds for each class—land, marine animal, and sky, respectively—*I_segNGMR_* is the segmented image using the *NGMR* index; this image can be used for photo-identification of marine animals by the dorsal fin, its skin, and other characteristics. In the case of using the *NBMG* index, the *I_segNGMR_* and *NGMR_morphCj_* in Equation (5) change to *I_segNBMG_* and *NBMG_morphCj_*, respectively, and the new thresholds are *T_NBMGC_*_1_ = 0.4491, *T_NBMGC_*_2*a*_ = 0.4492, *T_NBMGC_*_2*b*_ = 0.5898, and *T_NBMGC_*_3_ = 0.5899. During experimental simulations, we note that the marine animal and sea share common information that does not allow for good segmentation of each region. This is caused by the fact that the skin of the marine animal, with the reflection of sunlight, makes a mirror effect with the sea. For this reason, the proposed method uses the data of sea and marine animals as a unique class denominated class marine animal to perform Equation (5).

#### 2.1.4. Step 4: Selection and Classification of Regions

A region is regarded as a neighborhood of connected pixels. Let us consider two pixels *p* and *q* ∈ *Z* × *Z*. The pixels *p* and *q* are considered to be connected if *q* is in the set *N_α_*(*p*), where *α* is a metric of length path. Therefore, pixel *p* is adjacent to pixel *q* if both pixels are connected. Then, the path between the pixels *p* and *q* in a region is a sequence of pixels ${p_{0},p}_{1},\;p_{2},\;\ldots ,\;p_{n}$ such that $p_{0}=p$, $p_{n}=q$, and $p_{i}$ is adjacent to $p_{i-1}$ for 1 ≤ *i* ≤ *α* [34]. We propose neighborhoods of length path *α* = 8 to obtain the region where the marine animal is in the segmented image *I_segNGMR_* or *I_segNBMG_*.

The data training for the regions to be classified is obtained from the original images of the dataset. For this purpose, let us consider classes *C*_1_, *C*_2_, …, *C_k_* for 1 ≤ *j* ≤ *k*, *j* ∈ *C_j_*, where *k* is the number of classes; in our case, *k* = 3, taking into account that the sky, marine animal, and land are the classes to be classified [35,36,37]. The *SURFs* (Speeded-Up Robust Features) algorithm [35,38] is used to classify the proposed classes. The SURFs descriptor is a method for computing distinctive, invariant local features fast [38,39]. This is an efficient implementation of the SIFT (Scale Invariant Feature Transform) descriptor and is faster than a SIFT descriptor for variations in deformations such as image rotation, image blur, light changes, scale changes, JPEG compression, and viewpoint changes [39,40]. The SURFs descriptor, using a determinant of the Hessian matrix, detects feature points [39,41] and consists of two main parts [39,40]: a detector and a descriptor. The detector reduces the computation time significantly using an integral image, Hessian matrix-based interest points, scale-space representation, and interest point localization [39]. For a descriptor, each interest point searched by the detector has to carry its own indicator to assign invariance to the interest points [39]. When deformations occur in an image, the interest point descriptors can be utilized to look for correlations between the original image and the transformed image [39]. Also, the SURFs descriptor performs robustness against variations in scale, rotation, and illumination. These characteristics are especially relevant in images captured during sightings, where the acquisition conditions are uncontrolled and the quality of images varies due to the use of heterogeneous digital cameras. Although deep learning-based approaches like YOLO have demonstrated high performance in object detection [42], their effectiveness depends on large volumes of labeled data and specialized computational infrastructure, presenting risks of overfitting in scenarios with limited datasets [43]. The set of 1000 images is insufficient to representatively capture the variability of the marine environment, which limits the generalizability of deep learning models. While SURFs allow detection based on local characteristics, suitable for locating marine animals without requiring supervised training, enabling detection is a relevant aspect in research applied to the monitoring of fauna in natural environments [44]. For these reasons, the SURFs algorithm is applied to each training image for the three classes to be classified in the following way,

(6) $$P_{C_{j}}=\;SURF\left({Img}_{C_{j}}\right),\;\mathrm{and}\;W_{c_{j}}=\sum_{\iota \in C_{j}}^{N}P_{C_{j}}(\iota )\mathrm{for}\;1\leq \iota \leq N$$

where *P_Cj_* are the descriptors of scale-invariant local characteristics [33,38] of each training image, *W_Cj_* is the sum of the descriptors of the classes or the weights for each one of the classes, *N* = 100 is the number of training images for each class, and *Img_Cj_* (*Img_C_*_1_, *Img_C_*_2_, and *Img_C_*_3_ are the training images for sky, marine animal, and land, respectively) are images of the training classes. For example, the weight of the set *W_C_*_1_ is the sum of the descriptors *P_C_*_1_ of the 100 sky images *Img_C_*_1_ to which the *SURFs* algorithm is applied.

In the same way, the class of regions where the marine animal is possibly found in the segmented image is obtained in the following way,

(7) $$P_{RegionSelect}=\;SURF(I_{segNGMR})$$

where *I_segNGMR_* can be change for *I_segNBMG_*.

Finally, the maximum value *R_j_* is calculated, which indicates the class that belongs to the region obtained in the segmented image,

(8) $$max\left\{R_{j}:1\leq j\leq k\right\}\mathrm{with}\;R_{j}=\frac{M_{j}}{W_{C_{j}}},\;1\leq j\leq k$$

where $M_{j}=\sqrt{{\left(P_{RegionSelect}-P_{C_{j}}\right)}^{2}}$ is the Euclidean distance between the data region of the segmented image and the data of each region of the training images, that is, *M_j_* provides the value of coincidence where possibly the marine animal is located.

This classification algorithm provides the right location of the marine animal in the sighting image that can be used for tracking individual animal applications.

## 3. Results

Experimental results for the segmentation and location of marine animals are presented in this section.

**Figure 1:**
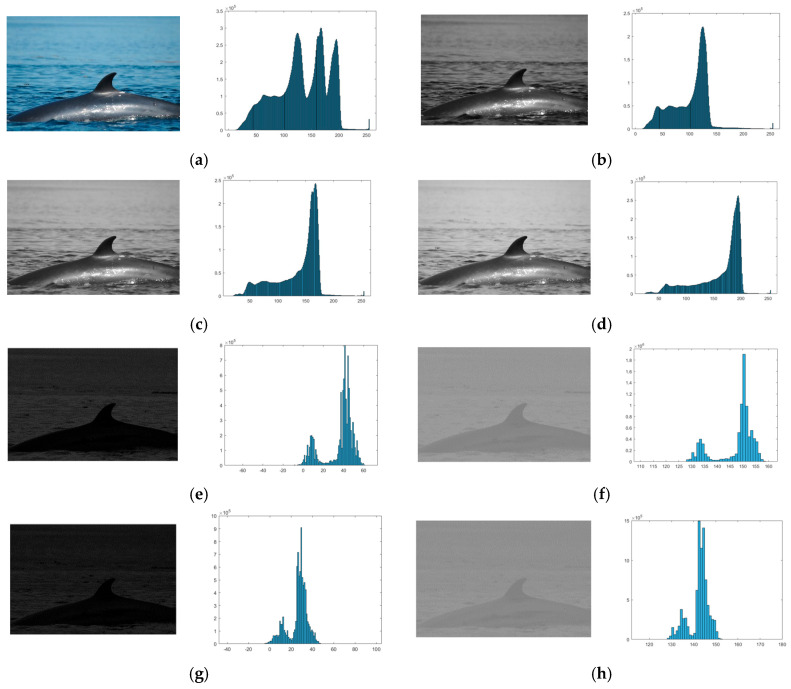
Images and histograms: (**a**) Original RGB image; (**b**) *R* image; (**c**) *G* image; (**d**) *B* image; (**e**) *GMR* image; (**f**) *NGMR* image; (**g**) *BMG* image; (**h**) *NBMG* image.

Figure 1 shows the images and histograms for the *R*, *G*, and *B* channels and the *GMR*, *NGMR*, *BMG*, and *NBMG* color indexes for a sighting image. In the histogram of each channel, the distribution of data visually makes it difficult to separate the information related to each region that makes up the original image. The histograms of the *GMR* and *BMG* color indexes show a distribution of data that allows easily separating the regions contained in the image. These histograms present a range of negative and positive values, which indicate the amount of red color compared to the amount of green color and vice versa or blue color compared to the amount of green color and vice versa, eliminating similar reflectance between the channels. The *NGMR* and *NBMG* images provide image enhancement by making the intensity between different elements in the image more distinguishable compared to the *GMR* and *BMG* images.

**Figure 2:**
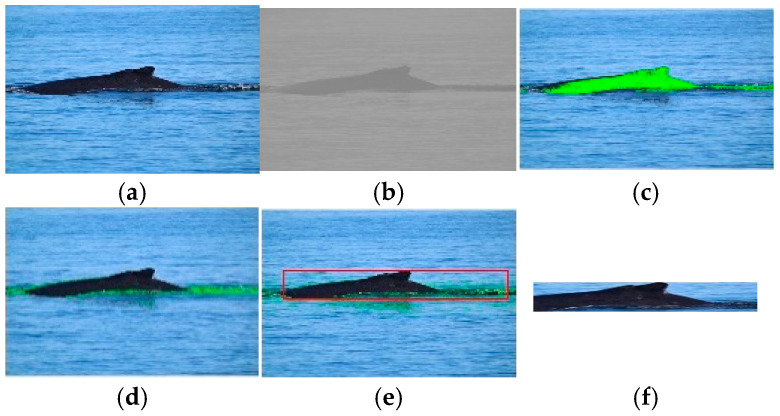
Visual results of segmentation and location of marine animal: (**a**) original image; (**b**) *NBMG* image; (**c**) segmented image *I_segNBMG_*; (**d**) segmented image *I_segNGMR_*; (**e**) marine animal location; (**f**) region of interest. The green color is the segmentation of marine animal. The red rectangle is the location of marine animal.

Figure 2 depicts a marine animal segmentation and location using the *NGMR* and *NBMG* indexes in an image of the dataset (see Figure 2a). In Figure 2b, the *NBMG* image and its corresponding segmented image, *I_segNBMG_*, are presented. In Figure 2c, we omit the *NGMR* image because this does not provide relevant information to obtain a segmentation of marine animals, as can be seen in the segmented image *I_segNGMR_* of Figure 2d. Figure 2e shows the marine animal location in a rectangle of red color in the image; this represents the marine animal location, and finally, the region of interest (ROI) is depicted in Figure 2f.

We investigate the options to utilize the *NBMG* and *NGMR* to obtain the best segmentation and/or the best location. After numerous simulations with these indices in the images of the dataset, we decided to improve the results of using the *NBMG* and *NGMR* indices independently by using both indices (*NGMR* + *NBMG*) in the same methodology.

**Figure 3:**
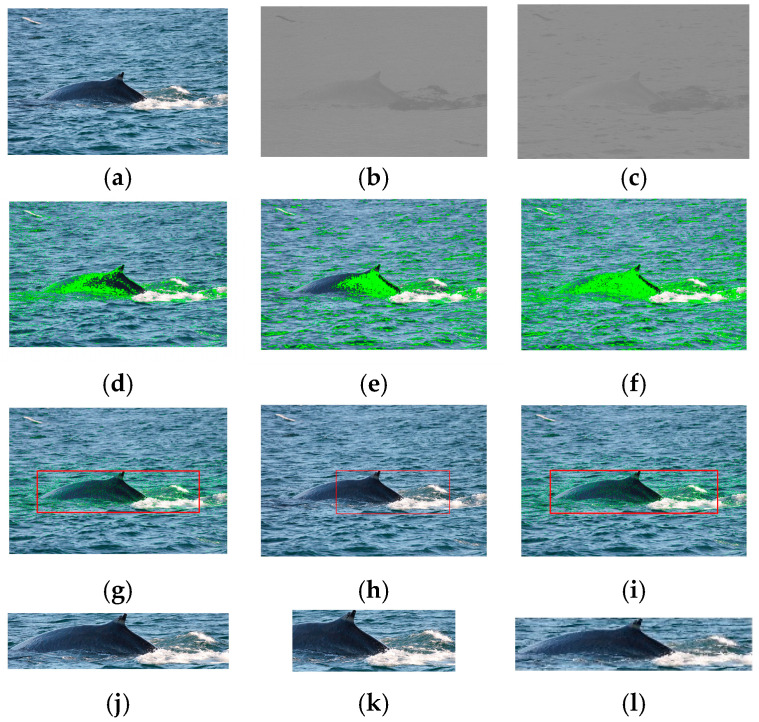
Visual results of segmentation and location of marine animals using the proposed indexes: (**a**) original image; (**b**) *NGMR* image; (**c**) *NBMG* image; (**d**) segmented image *I_segNGMR_*; (**e**) segmented image *I_segNBMG_*; (**f**) segmented image with both indexes; (**g**) location of marine animal with *NGMR*; (**h**) location of marine animal with *NBMG*; (**i**) location of marine animal with both indexes; (**j**) region of interest with *NGMR*; (**k**) region of interest with *NBMG*; (**l**) region of interest with both indexes. The green color is the segmentation of marine animal and the red rectangle is the location of marine animal.

Figure 3 depicts the visual results of segmentation and location of a marine animal (see Figure 3a) using *NGMR*, *NBMG*, and *NGMR* + *NBMG* indexes. Figure 3b,c present the *NGMR* and *NBMG* images, respectively, where one can see that these images contain different details of the marine animal. Consequently, Figure 3d,e provide different segmentation results of the marine animal, but Figure 3f shows a better segmentation in comparison with the other ones. This is because the algorithm takes into account the data of both indices. Figure 3g–i present the location of the marine animal in the image in a rectangle of red color. These results reveal that the three proposals can obtain the location of marine animals in the image, but do not provide the same results, as can be seen in the ROIs of Figure 3j–l. This is because the location depends on the segmentation result. Finally, Figure 3 shows an example where the *NGMR* and *NBMG* do not provide a good segmentation, but the method can obtain the location of the marine animal. When the *NGMR* + *NBMG* indexes are used, they can improve the segmentation results in comparison to when the indexes are used independently, and consequently, the best location is obtained.

**Figure 4:**
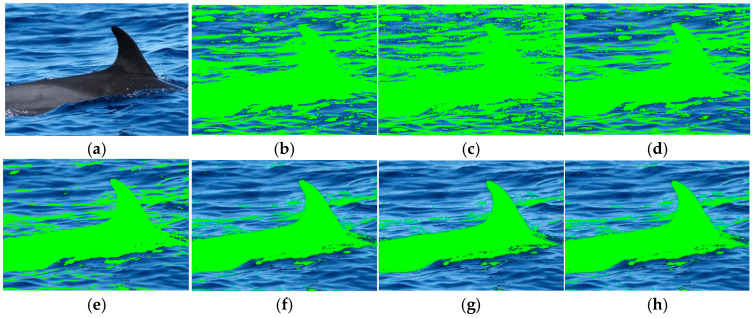
*NGMR* + *NBMG* segmentation results using the thresholds obtained in Table 1 for different numbers of images used as training images: (**a**) original image; (**b**) segmentation with 5 images; (**c**) segmentation with 10 images; (**d**) segmentation with 20 images; (**e**) segmentation with 40 images; (**f**) segmentation with 60 images; (**g**) segmentation with 80 images; (**h**) segmentation with 100 images. The green color is the segmentation of marine animal.

Additionally, the proposed method can resolve the limitations of large training data presented in the deep learning approaches to find the appropriate thresholds to perform an optimal segmentation. Table 1 presents the obtained thresholds by using 5 and 10. Furthermore, 20, 40, 60, 80, and 100 images of each class were randomly selected as training images according to the procedure shown in Section 2.1.3. From Table 1, the threshold values found for *NGMR* and *NBMG* vary in the second significant number in most cases, ensuring that the proposed method provides a solution when there is not sufficient data to realize a large training dataset. Figure 4 shows the *NGMR* + *NBMG* segmentation results for an image of the happywhale dataset using the thresholds obtained in Table 1 for different numbers of images used as training images. From Figure 4, the segmentation results reveal that the use of all proposed thresholds permits the segmentation of marine animals, but the best results are given when the thresholds are presented for the training with 40, 60, 80, and 100 images.

During the realization of these tests, we utilize images of the dataset that do not have good environmental conditions when the image is acquired, or the parameters of acquisition are inappropriate when the image is captured, or the content of the image does not have sufficient representative elements, among others. During the tests in these images, the experimental results reveal that the proposed method provides good results in terms of segmentation and location in most cases. For these reasons, it is expected that the proposed method can improve the performance results if the tested images take into account the issues described above, for example, using acquired images with controlled conditions or a preprocessing stage to enhance the characteristics of the tested images.

**Figure 5:**
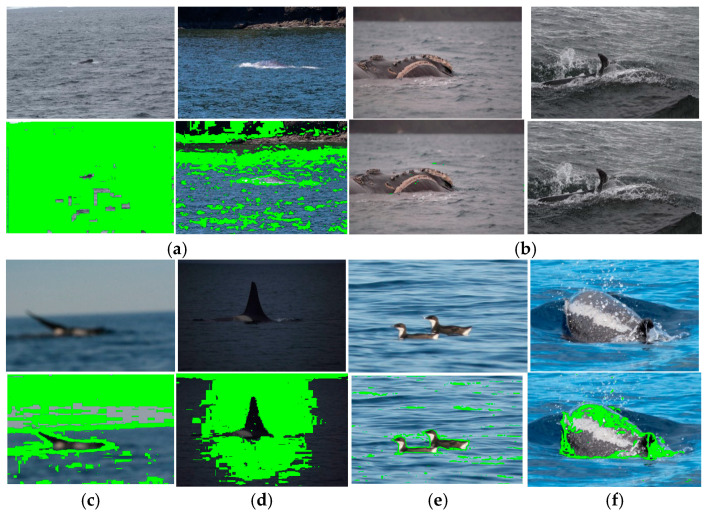
Visual criteria for discarding sighting images and their segmentations: (**a**) images where the marine animal is very small within the image; (**b**) images where the marine animal does not show all its characteristics completely; (**c**) blurry images; (**d**) low-illumination images; (**e**) images with other species; (**f**) images that do not show the characteristics of the marine animal due to the angle of capture of the camera. The green color is the segmentation of marine animal.

Normally, the sighting images of marine animals have common elements such as the sea, land, and sky in most cases. Each of the sighting images features intrinsic challenges like those already mentioned above. Figure 5 shows some images where the proposed method could not obtain the complete segmentation and consequently the corrected marine animal location for tracking. In Figure 5a are presented images where the marine animal is very small within the images; Figure 5b presents images where the marine animal does not show all its characteristics completely; Figure 5c depicts a blurry image; Figure 5d presents an image with low illumination; Figure 5e contains other species; and Figure 5f does not show the characteristics of the marine animal due to the angle of capture of the camera. The images of Figure 5 and other ones with the same characteristics are included in the experimental results presented in this section. About the segmentation results, one can see in Figure 5 that in the case of these images, the segmentation algorithm is not able to obtain the marine animal segmentation well; for this reason, the classification of marine animals could be incorrect, and consequently, the location and tracking are not realized. For example, in Figure 5a,c,d, the segmentation algorithm obtains as a segmentation result almost all regions of the images; these results cannot classify the marine animal correctly; therefore, the process of location is not provided. In Figure 5b, the segmentation algorithm cannot obtain any segmentation; consequently, the location is not available, and Figure 5e,f show a correct segmentation of the birds and the marine animal, respectively, but the classification algorithm considers that the birds are marine animals and continues with the location. This is an incorrect result, and in the case of a marine animal, this algorithm falls in the correct recognition of a marine animal, but the location is not provided. Therefore, in the case of the image of the birds, the segmentation algorithm provides only one segmentation of the two birds; that is, the algorithm is not able to distinguish when there are two birds. In the case, of multiple marine animals appeared simultaneously in an image, the segmentation algorithm only performs one segmentation, this is because the proposed model does not quantify the number of individuals presented in the image and these are not processed independently, instead, the algorithm considers the scene as a single entity, and the classification algorithm takes the segmentation of multiple marine animals as only one animal and the recognition performance is realized as this is as only one marine animal.

To evaluate the performance of the proposed method, 1000 images are chosen randomly from the happywhale dataset. The *Specificity*, *Accuracy*, *Precision*, *Recall*, and *F-measure* [37,42] are used to measure the performance of the proposed algorithm in the segmentation + location of the marine animal in the images of scientific sightings.

*Specificity*. This metric shows the probability that the region of interest is not a marine animal since there is no marine animal in the selected region of interest,

(9) $$Specificity=\frac{TN}{\left(TN+FP\right)}$$

*Accuracy.* This metric measures the output of the classifier that correctly predicted the total set of samples analyzed,

(10) $$Accuracy=\frac{\left(TP+TN\right)}{\left(TP+TN+FP+FN\right)}$$

*Precision* or positive predictive value is the proportion of observations from the positive class correctly classified as positive,

(11) $$Precision=\frac{TP}{\left(TP+FP\right)}$$

*Recall* or true positive rate is the proportion of correctly classified positives,

(12) $$Recall=\frac{TP}{\left(TP+FN\right)}$$

*F-measure* or *F*_1_ represents the total effectiveness of a classifier,

(13) $$F-measure=\frac{\left(2\;TP\right)}{\left(2TP+FN+FP\right)}$$

In our study case, *TP* (True Positive) shows that the segmentation and location of the marine animal are correct, *TN* (True Negative) indicates that the marine animal is not located and is not indeed located, *FP* (False Positive) indicates that the marine animal is found, but it is not a marine animal, and *FN* (False Negative) shows that the marine animal is not found, but it is indeed a marine animal.

The confusion matrix obtained with the proposed method using the *NGMR*, *NBMG*, and *NGMR* + *NBMG* indexes is depicted in Table 2. Comparing the *NGMR* and *NBMG* classification results, the best classification is in favor of *NGMR*, with +66 positive samples in comparison with the *NBMG* classification. When the three proposals are compared, the classification results reveal that the *NGMR* + *NBMG* outperforms the classification results with +36 and +102 samples classified positively in comparison with *NGMR* and *NBMG*, respectively. These results are in accordance with the visual results of Figure 2 and Figure 3.

Table 3 presents the performance of the proposed method for the segmentation + location of the marine animal in sighting images. The *Precision* results reveal that the *NGMR* + *NBMG* provides the best results in comparison with *NGMR* (−0.06) and *NBMG* (−0.21), respectively. In the same way, the *Specificity*, *Accuracy*, and *F-measure* have the same compartment as the *Precision*; that is, the performance values of *NGMR* + *NBMG* outperform the results of *NGMR* and *NBMG*. In the case of Recall, the best result is in favor of *NGMR* with +0.02 in comparison with *NGMR* + *NBMG*.

The performance of the proposed method (see Table 3) has been compared with other methodologies that use the humpback whale images of the happywhale database. A deep learning approach based on the ArcFace classification head provides a level of precision below 0.84 [45] in comparison with the precision of 0.98% of the proposed *NGMR* + *NBMG*; and the DeepSense algorithm uses a series of convolutional neural networks to obtain an accuracy of 87% [46]. In this case, the proposed *NGMR* + *NBMG* algorithm achieves the same accuracy. An animal photo-identification using deep metric learning and network analysis achieves a specificity close to 80% in comparison with the specificity of the proposed NGMR + NBMG algorithm, which achieves 96% [47], and an automatic identification system for humpback whales using convolutional neural networks performs an accuracy of 78.5% in comparison with the 87% provided by the proposed NGMR + NBMG algorithm, demonstrating a significant improvement over previous methodologies based on traditional CNNs [24].

**Figure 6:**
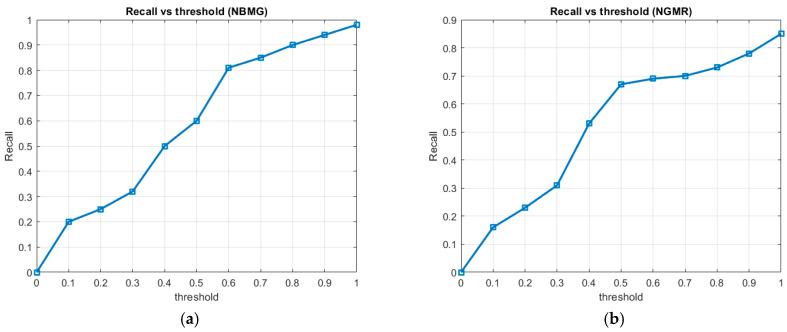

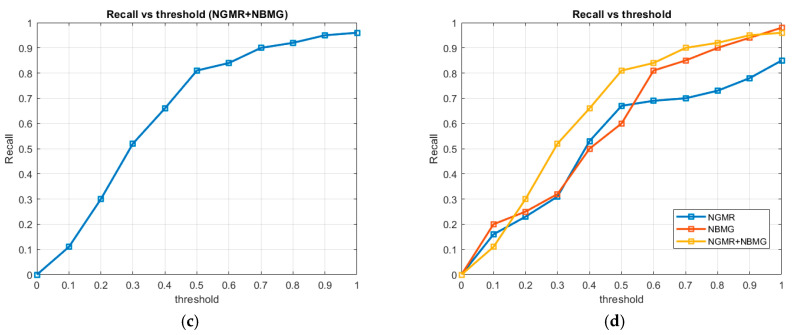
Performance ROC curve (graphs of *Recall* vs. threshold) for different approaches: (**a**) *NBMG*; (**b**) *NGMR*; (**c**) *NGMR* + *NBMG*; (**d**) the three approaches compared.

We also utilize the receiver operating characteristic (ROC) curves to illustrate the performance of a classifier by plotting the *Sensitivity* (*Recall*) and *Specificity* [48,49]. The total area under the ROC curve is a single index for measuring the performance of a test. The performance of different image classification algorithms is analyzed, and three normalization approaches were tested: *NGMR*, *NBMG*, and *NGMR* + *NBMG*. The study used the images from the happywhale dataset. This dataset considered images containing marine animals and also included images without any marine animals. The proposed sample set has a total of 1000 images taken randomly. The objective was to measure the performance of the different approaches proposed in this research in terms of *Recall*. This is defined as the percentage of images that actually contained marine animals and were correctly identified by the models. For each normalization approach, performance graphs were obtained showing the relationship between the threshold (threshold value) and the *Recall*, allowing us to observe how the *Sensitivity* performance of the approach (normalization of the color index) varies at different decision levels. Figure 6 depicts the graphs of *Recall* vs. threshold for the proposed normalization approaches.

The *NBMG* approach (see Figure 6a) presents an ascending curve in the Recall values when the threshold increases, which indicates a progressive improvement in the detection of true positives. The *NGMR* (see Figure 6b) shows a similar trend to the previous approach, although with a steeper slope at high threshold levels, reaching a *Recall* close to 0.85, indicating a higher number of true positives. For the *NGMR* + *NBMG* (see Figure 6c), the combination of both normalizers offers superior performance, with sustained improvement in *Recall* across all threshold levels. This synergy suggests greater robustness of the model in identifying marine animals, even under low-presence conditions. The qualitative and quantitative comparison of the results obtained in Figure 6a–c suggests that the combination of *NGMR* + *NBMG* provides superior *Recall* performance, especially in scenarios with high thresholds (see Figure 6d). This indicates that integrating both normalization approaches can improve the ability to correctly identify images containing marine animals.

The proposed method employs the RGB color space, this has been compared with other well-known state-of-the-art segmentation algorithms [50], that are based on the RGB color space such as the histogram thresholding Fuzzy C-means (HTFCM) [51], the Improved Ant Colony-Fuzzy C-means Hybrid Algorithm (IAFHA) [52], and the stochastic nonparametric expectation maximization (SNEM) [53], and other ones based on the CIELab color space such as the penalized inverse expectation maximization (PIEM) [54] and the segmentation by clustering then labeling (SCLpost) [55] algorithms.

**Figure 7:**
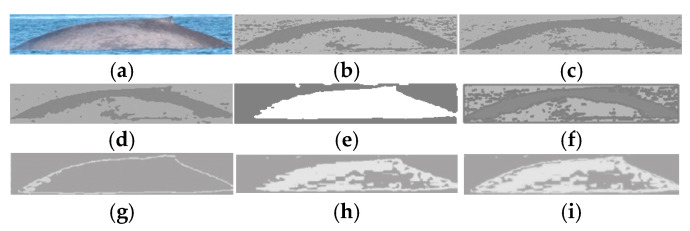
Segmentation results on sighting image using different segmentation methods: (**a**) original image; (**b**) HTFCM; (**c**) IAFHA; (**d**) SNEM; (**e**) PIEM; (**f**) SLCpost; (**g**) proposed NGMR; (**h**) proposed NBMG; (**i**) proposed NGMR + NBMG.

Figure 7 depicts the comparative segmentation results in a sighting image. We observe that our proposed method can segment the image well in comparison with other methods; these results can be utilized in some cases in the photo-identification of marine species, such as dorsal and caudal fins. It is noted that the comparative methods use more complex algorithms than the proposed method; this implies that the proposed method provides less processing time to compute the marine animal segmentation in comparison with the methods used as comparison. Also, although the proposed method is based on traditional RGB processing concepts, the proposed method can achieve better visual segmentation results in comparison with the comparative methods that use learning in most cases. In the case of Figure 7, the proposed method is only surpassed by the PIEM.

To demonstrate that the proposed method can be used in real-time marine animal tracking, the processing time is computed using a laptop with an Intel^®^ Core™ i7-10750H CPU @ 2.60 GHz processor, 16 Gbytes RAM, and MATLAB^®^ 2018b. Images of 645 × 376 and 3600 × 2431 pixels were tested, obtaining an average of 0.33 s and 7.0 s, respectively. It is noted that the processing time can be reduced if the proposed algorithm is run on digital signal processing modules.

**Figure 8:**
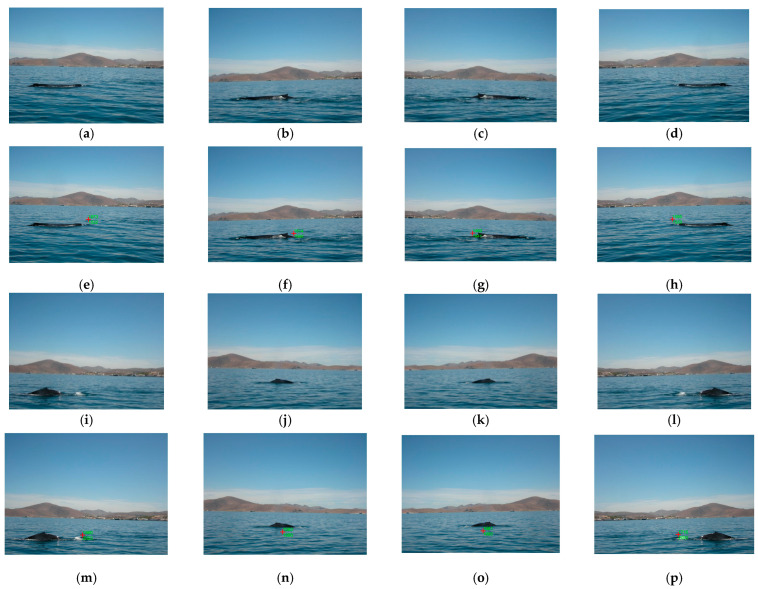
Location results on a simulated video sequence: (**a**–**d**,**i**–**l**) are the original frames; (**e**–**h**,**m**–**p**) are the location coordinates results of (1673, 1532), (1814, 1840), (1460, 1841), (1595, 1533), (1560, 2053), (1583, 1990), (1681, 1990), and (1716, 2050) for each one of the original images, where the coordinates (0, 0) are located in the top left of the image.

Additionally, to clarify the process of marine animal location for tracking, we simulate a video sequence with some images taken from the dataset where the marine animal is found in a different position in each image. The bounding box used for this purpose (see Figure 2 and Figure 3) is changing for a red mark “+” to show the coordinates (*x*,*y*) in green color for each frame where the marine animal is located. Figure 8 presents the frames of the simulated video sequence used in this test, where the location is shown in each frame with its respective coordinates.

During the realization of this test, two issues are discussed:

1. The coordinates of the marine animal location in the real world can be obtained by transforming the marine animal location coordinates found in each frame, utilizing a computer vision system, and then using these real-world coordinates to move a smart camera to track the specific animal target. In our case, this process is not possible to realize because the dataset used only contains images, and we do not have access to smart cameras with a coordinate mechanism to provide the tracking.
2. The proposed algorithm does not use the voxel processing concept or temporal information between video frames by using the information of frame (*t* − 1) and/or frame (*t* + 1) to process the actual frame (*t*); that is, previous or successive frames are not taken into account. The use of various frames improves the performance results because during the tracking process, the algorithm utilizes more information about the movement of the marine animal, which, when utilizing only one frame, is limited by the fact that the processing time is increased. Therefore, the proposed location algorithm for tracking by using one video frame could be limited to solving problems of rapid changes in environmental conditions and/or illumination and other types of problems related to the location of marine animals, such as occlusion, deformation, and rapid movements. These rapid changes could not provide a good segmentation; if the segmentation is not correct, the location of the marine animal is not provided, and the tracking fails. We are sure of the limitations of the proposed algorithm described above, but one of the contributions of the proposed algorithm is the real-time processing of a video frame in any device; the precision and/or accuracy could be decreased.

Finally, the proposed method is simple, efficient, and feasible for marine animal applications; it does not require a lot of computing resources, and its implementation is straightforward on any device and can provide a real-time solution to process marine animal video frames.

## 4. Discussion

The proposed segmentation + location method is easy to implement in any device because it computes simple calculus, but this produces advantages and disadvantages. The use of *NGMR*, *NBMG*, and *NGMR* + *NBMG* indexes helps to obtain information about the colors in the sighting images to properly segment the marine animal. In the case of photo-identification, the main issue to solve is the common information (colors) that the sea and the marine animal share, which makes it difficult to segment, as can be seen in Figure 1, Figure 2, Figure 3 and Figure 7. The proposed method utilizes a simple supervised classifier to segment the marine animal with sufficient information to perform a photo-identification. The issue of common information could be solved with a robust classifier based on FCM, neural networks, or deep learning to obtain more accurate data for the segmentation, but the processing time can increase, and this is not the objective of the paper, which is to provide a simple method with low computing resources for real-time applications. Additionally, a preprocessing stage can be implemented to enhance the characteristics of the regions presented in the sighting image to improve the segmentation + location results.

The comparative segmentation results reveal that the proposed method can obtain similar results in comparison with other methods, but the difference is that the comparative methods use complex techniques based on FCM, Ant Colony, nonparametric expectation maximization, and labeling that are more robust in comparison with the simple classifier utilized in the proposed method. This is an advantage because the proposed method provides less calculus than the other ones.

The standard SURFs method is easy to implement, and this provides the location of the marine animal within the sighting image. This algorithm provides good results but depends on the data of segmented marine animals.

The performance of the proposed method in the segmentation + location of the marine animal shows a precision up to 0.98 and an average-high accuracy of 0.99; these demonstrate that the proposed method is the right option for the segmentation + location of marine animals.

The proposed method could be used in marine animal tracking in real-time applications because images of 645 × 376 pixels or less can be processed at almost two frames or more per minute with the laptop used in this research. It is obvious that with a signal processing device, the time could decrease drastically.

## 5. Conclusions

In this paper, a novel algorithm for the first stage of marine species photo-identification and location methods is presented. In the case of photo-identification, a color index-based thresholding method is proposed for marine species segmentation. The proposed method can resolve the limitations presented in the deep learning approaches. This method does not require a large amount of training data to find the appropriate thresholds to perform an optimal segmentation. This method utilizes the proposed *NGMR*, *NBMG*, and *NGMR* + *NBMG* indexes, which allow different segmentation results where the best segmentation is found using *NGMR* + *NBMG*. The proposed segmentation method can be utilized in different marine species by changing the threshold in an easy way and without large training images. For marine species location, a *SURFs*-based supervised classifier is used to provide the location of marine animals in the image. This is achieved by the processing of a segmentation image. Experimental results in the happywhale dataset show a precision for the segmentation + location of the marine animal of 0.77, 0.92, and 0.98 using the *NBMG*, *NGMR*, and *NGMR* + *NBMG*, respectively. The proposed method with *NGMR* + *NBMG* indexes provides the best results because it combines the color information of both indexes, allowing more color information from each one of the regions contained in the image. The performance results can be improved with other datasets where the sighting images have better environmental conditions, parameters of acquisition, and a complete representative marine animal when the image is acquired, in comparison with the images of happywhale dataset. In our case, the happywhale dataset is used to demonstrate the robustness of the proposed method in the task of segmentation and location for tracking. The proposed method is compared with other methodologies based on deep learning that use the happywhale dataset, achieving an improvement in the precision performance, and with the same accuracy. The proposed method could be used in real-time marine species tracking with a processing time of 0.33 s for images of 645 × 376 pixels using a standard PC. This time can be reduced if the proposed algorithm is running on DSPs or FPGAs. Finally, the proposed method is simple, efficient, and feasible for marine species applications. This does not require a lot of computing resources, and its implementation is straightforward on any device and could provide a real-time solution to process marine animal video frames.

## Figures and Tables

**Table 1:** Thresholds obtained with the use of different numbers of images for each class as training images.

Images	NGMR				NBMG			
	*T_NGMRC_*_1_	*T_NGMRC_*_2*a*_	*T_NGMRC_*_2*b*_	*T_NGMRC_*_3_	*T_NBMGC_*_1_	*T_NBMGC_*_2*a*_	*T_NBMGC_*_2*b*_	*T_NBMGC_*_3_
5	0.3905	0.3906	0.6211	0.6212	0.4022	0.4023	0.6172	0.6173
10	0.4335	0.4336	0.5977	0.5978	0.4101	0.4102	0.6211	0.6212
20	0.4413	0.4414	0.5977	0.5978	0.4101	0.4102	0.6133	0.6134
40	0.4374	0.4375	0.5820	0.5821	0.4687	0.4688	0.6016	0.6017
60	0.4491	0.4492	0.6016	0.6017	0.4452	0.4453	0.5898	0.5899
80	0.4257	0.4258	0.5781	0.5782	0.4608	0.4609	0.5820	0.5821
100	0.4608	0.4609	0.5781	0.5782	0.4491	0.4492	0.5898	0.5899

**Table 2:** Confusion matrix.

	NGMR		NBMG		NGMR + NBMG	
Classifier Result	Positive	Negative	Positive	Negative	Positive	Negative
Positive	527	48	461	136	563	13
Negative	93	332	104	299	119	305
Total	620	380	565	435	682	318

**Table 3:** Performance of proposed method.

Metric	NGMR	NBMG	NGMR + NBMG
Precision	0.92	0.77	0.98
Specificity	0.87	0.69	0.96
Accuracy	0.86	0.76	0.87
F-measure	0.88	0.79	0.90
Recall	0.85	0.82	0.83

## Data Availability

The data will be available from the authors upon request.
